# Supplementary material for: Two for One—Combined Morphologic and Quantitative Knee Joint MRI Using a Versatile Turbo Spin-Echo Platform
Source: Diagnostics (Basel). 2024 May 8;14(10):978. doi: 10.3390/diagnostics14100978 (PMC11120432; doi:10.3390/diagnostics14100978)
Supplement: Supplementary file 1 [file diagnostics-14-00978-s001.zip › diagnostics-2940637-supplementary.pdf]

Supplementary Material

Supplementary Figure S1

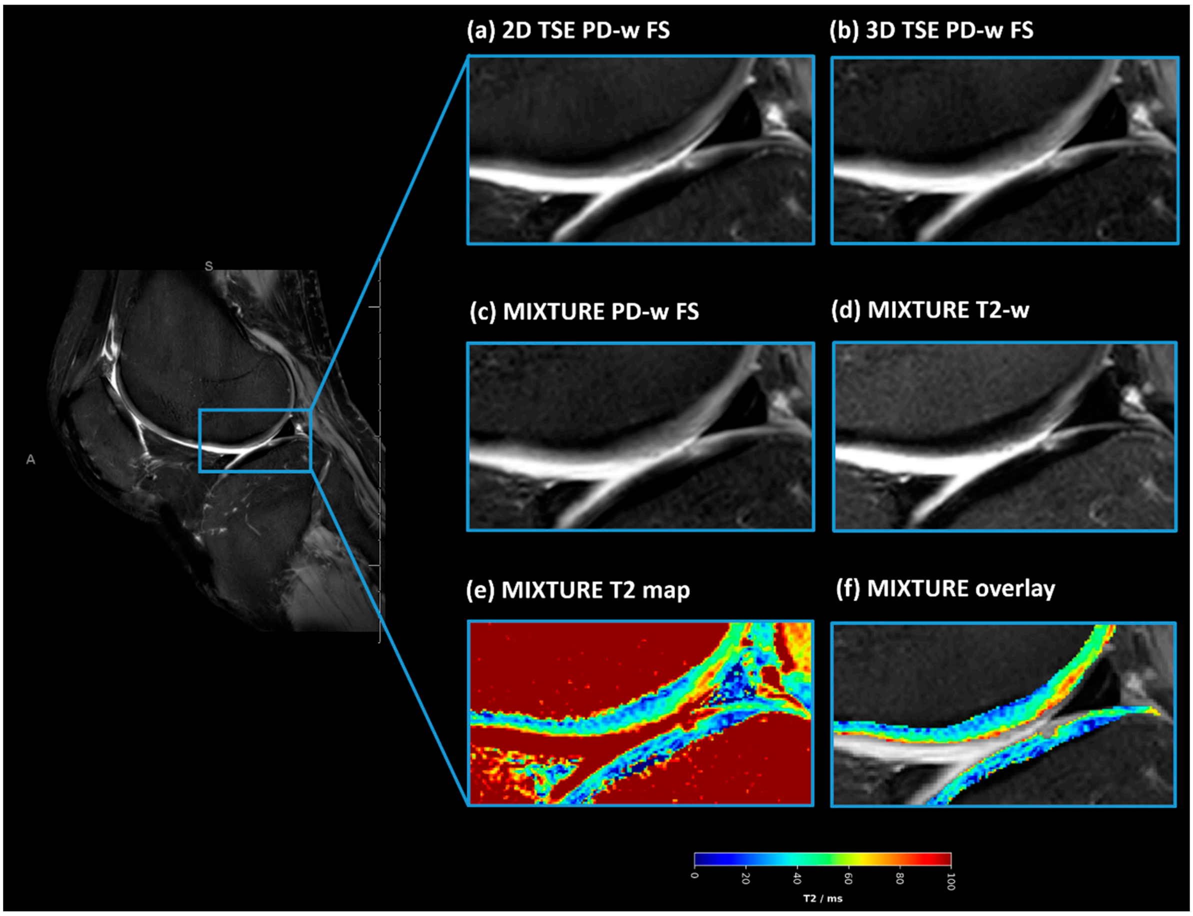

Figure S1: Close-up of the central weight-bearing region (proton density-weighted fat-saturated MIXTURE and corresponding reference sequences).
The blue box indicates the zoomed-in area. Figure organization, specimen, and slice as in **Figure 2**.

Supplementary Figure S2

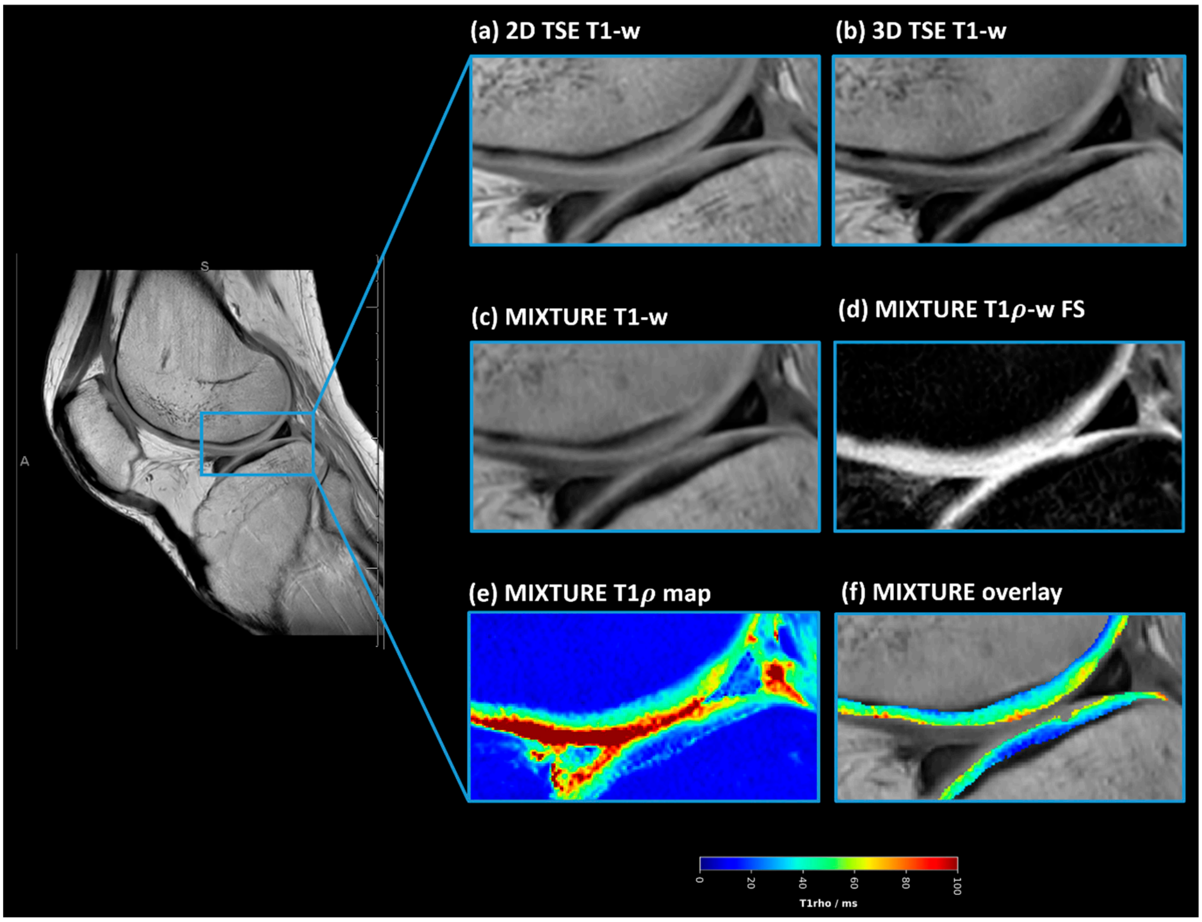

Figure S2: Close-up of the central weight-bearing joint region (T1-weighted MIXTURE and reference sequences).

The blue box indicates the zoomed-in area. Figure organization, specimen, and slice as in **Figure 4**.
